# Efficacy and safety of oral compared with intravenous tranexamic acid in reducing blood loss after primary total knee and hip arthroplasty: a meta-analysis

**DOI:** 10.1186/s12891-018-2358-2

**Published:** 2018-12-03

**Authors:** Xiaozhen Han, Guiqing Gong, Naili Han, Mei Liu

**Affiliations:** 1 Department of Orthopedics, Jinan Zhangqiu District Hospital of Traditional Chinese Medicine, No. 1463 Xiushui Street,Mingshui subdistrict office, Zhangqiu district, Jinan, 250200 Shandong China; 2 Clinical Laboratory, Jinan Zhangqiu District Hospital of Traditional Chinese Medicine, No. 1463 Xiushui Street,Mingshui subdistrict office, Zhangqiu district, Jinan, 250200 Shandong China; 3 Department of Intensive Care Unit, Wuhan No.1 Hospital, No.215 Zhongshan Ave, Wuhan, 430022 Hubei China

**Keywords:** Tranexamic acid, Total hip arthroplasty, Total knee arthroplasty, Meta-analysis

## Abstract

**Background:**

Tranexamic acid (TXA) is an anti-fibrinolytic agent successfully preventing blood loss when using intravenously (IV) in total hip arthroplasty (THA) and total knee arthroplasty (TKA). An oral administration, which is available on blood sparing, has been reported exhibit profound cost-saving benefits. The aim of this meta-analysis is to investigate whether the administration of oral and intravenous tranexamic acid postoperatively has equivalent blood-sparing properties in these patients.

**Methods:**

The online electronic databases were searched for eligible literatures updated on September 2018. Studies assessing the effect between oral TXA and intravenous TXA (IV-TXA) in those undergoing TKA or THA were included. All the data were pooled with the corresponding 95% confidence interval (CI) using RevMan software. Based on the heterogeneity, we performed a systematic analysis to explore the overall results across the included studies.

**Results:**

Nine studies met our inclusion criteria. No significant differences were identified with regard to the Hb drop (SMD = − 0.03,95%CI = − 0.18–0.12, *P* = 0.67), total Hb loss (SMD = 0.10,95%CI = − 0.06–0.26, *P* = 0.24), total blood loss (SMD = − 0.00,95%CI = − 0.20–0.20, *P* = 1.00), transfusion rate (OR = 0.77,95%CI = 0.54–1.10, *P* = 0.14), DVT rate (OR = 0.58,95%CI = 0.19–1.75, *P* = 0.33), and length of hospital stay (SMD = − 0.05,95%CI = − 0.28–0.17, *P* = 0.63) between the oral groups and intravenous group.

**Conclusion:**

The blood-sparing efficacy of oral TXA is similar to that of the intravenous forms in the setting of THA and TKA. Considering the cost-benefit superiority and ease of administration of oral TXA, further studies and clinical trials are required to further identify the optimal administration for THA and TKA.

## Background

Patients who suffer from osteoarthritis, rheumatoid arthritis, fractures, or the knee or hip cancer often need total knee and hip arthroplasty (TKA and THA) [1]. Although TKA and THA have been identified as an effective management in improving physical function and relieving pain, these procedures can result in high risk of perioperative anemia, which can be associated with increasing morbidity and cost. Thus, a considerable number of patients require blood transfusions to prevent anemia [2, 3].

Finding effective methods for minimizing the risk of transfusion and perioperative blood loss have been a goal for surgeons to perform total TKA and THA [3, 4]. Surgeons have implemented numerous methods to achieve this goal, particularly the using of anti-fibrinolytic agents, such as tranexamic acid (TXA).

Tranexamic acid is a synthetic amino acid that functions by competitively inhibiting the activation of plasminogen, thereby inhibiting clot degradation [5], which has been identified as effectively reducing blood loss and the risk of adverse outcomes following THA and TKA [6–8].

Tranexamic acid can be administered intravenously (IV), topically (intra-articular), and orally. However, the optimal administration of TXA administration remains controversial [9–11]. Recently, several studies have been published to evaluate the effects of the TXA administration routes between oral and IV-TXA, while the efficacy and safety between two groups are still unknown [12, 13].

In this meta-analysis, we aim to explore whether oral TXA has an equivalent effect to intravenously in reducing blood loss in THA and TKA.

## Methods

### Search strategy

We performed the current meta-analysis based on the electronic databases: Pubmed, Embase, Cochrane library up to September 2018.The process was established to find all articles based on the MeSH terms and free key words: “Total knee replacement or arthroplasty” AND “Total hip replacement or arthroplasty” OR “tranexamic acid” AND “oral TXA” , AND “intravenous TXA”. Moreover, we identified full-text papers from reference materials for further evaluation.

### Eligibility criteria

Studies were included in the meta-analysis should meet the following criteria: [1] articles that enrolled total knee/hip arthroplasty patients treated with TXA; [2] the studies are designed as comparing TXA administered via intravenous (IV) and oral routes; [3] the outcomes of interest were efficacy (total blood loss, total hemoglobin (Hb) loss, Hb reduction, transfusion rate, length of hospital stay) and toxicity (incidence of deep vein thrombosis (DVT)), and with corresponding 95% CIs were provided; [4] the full-text papers were only included.

### Quality assessment

The quality of the search was performed separately by two reviewers. We use the New-castle-Ottawa Quality Assessment Scale recommended by the “Cochrane Intervention Manual Systematic Review”.

### Data extraction

Two authors independently extracted the relevant data from each trial. Disagreement was resolved by consensus. We extracted the main categories based on the following: first author family name, year of publication, study type, surgery, dosage of TXA patient number, age, and outcomes measures. We extracted the corresponding Std.Mean Difference (SMD) and odds ratios (ORs) with 95% confidence interval (95%CI) to describe the endpoints of interest.

### Statistical analysis

The Cochrane Collaborations have offered Review Manager Software (RevMan5.3) for statistical analysis.(Revman; The Cochrane collaboration Oxford, United Kingdom). The chi-square was used to assess the significance of heterogeneity [14]. I^2^ value larger than 50% suggested high degree of heterogeneity [15]. When there was high heterogeneity among studies, the randomed-effects model was used. Otherwise, the fixed effects model was used. A *P* value less than 0.05 was identified as statistically significant difference. Findings of our meta-analysis were shown in forest plots. The Begg test and the Egger test were conducted to evaluate publication bias.

## Results

### Study selection process

**Fig. 1:**
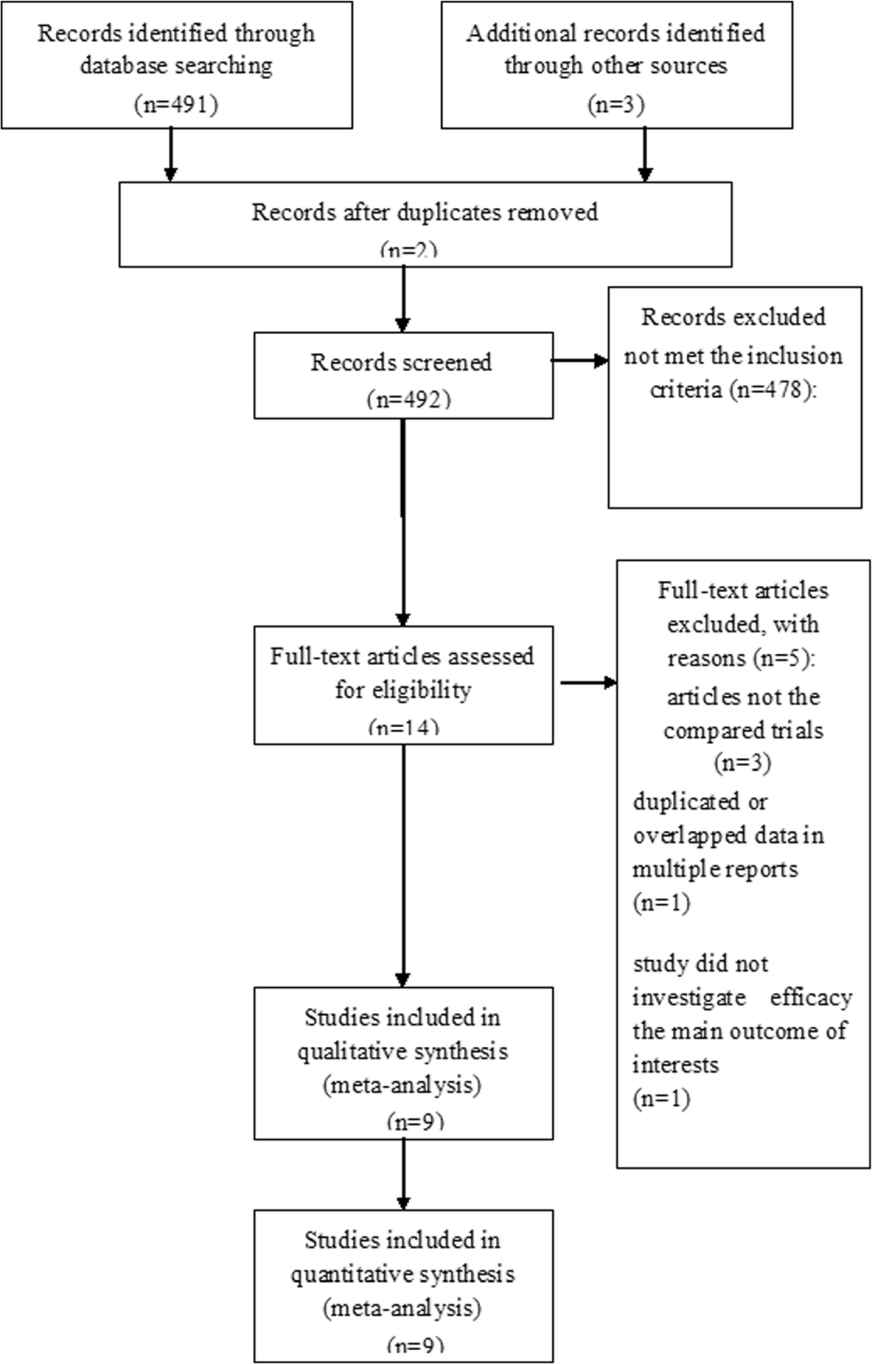
PRISMA flow chart of selection process to identify studies eligible for pooling

A total of 492 articles were considered possibly eligible. Based on the criteria described in the methods, 14 articles were further eliminated by reading the full articles, but some did not provide enough detail of outcomes of two approaches. Therefore, a final total of 9 studies [11, 16–23] with included (Fig. 1). Table 1 describes a brief description of these eligible studies. All included studies in this study were based on moderate to high quality evidence.

**Table 1:** the primary characteristics of the eligible studies in more detail

Studies (year)	Study type	Surgery	Treatment regimen (Dosage of TXA)		Treatment regimen (number)		Treatment regimen (Age)	
			oral	intravenous	oral	intravenous	oral	intravenous
Kayupovt, 2017	RCT	THA	1950 mg/kg	1000 mg	40	43	60	55
Yuan, 2017	RCT	TKA	40 mg/kg	20 mg/kg	140	140	63.2	63.7
Fillingham, 2016	RCT	TKA	1950 mg	1000 mg	34	37	62	63
Irwin, 2013	RCS	TKA and THA	25 mg/kg (maximum 2 g)	15 mg/kg (maximum 1.2 g)	302	2698	67.6	68.2
Zohar, 2004	RCT	TKA	4000 mg	10 mg/kg for 12 h	20	20	69	73
Cao, 2018	RCT	THA	2000 mg	20 mg/kg	54	54	55.7	55.7
Gortemoller, 2017	RCS	TKA and THA	1950 mg	1000 mg	165	165	67	68
Luo, 2017	RCS	THA	2000 mg	20 mg/kg	60	60	67.6	66.98
Wang, 2018	RCT	TKA	2000 mg	20 mg/kg	60	60	63.91	66.9

*TXA* tranexamic acid, *THA* total hip arthroplasty, *TKA* total knee arthroplasty, *RCT* randomized controlled trial, *RCS* retrospective cohort study

### Clinical and methodological heterogeneity

#### Pooled analysis of Hb drop between oral administration and intravenous administration

**Fig. 2:**
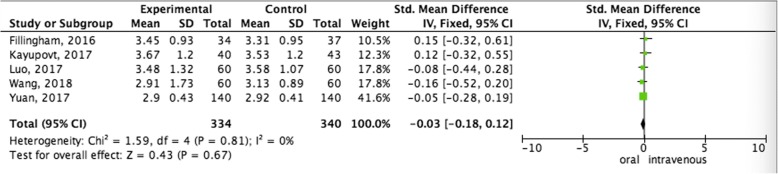
Pooled analysis of Hb drop between oral administration and intravenous administration

Pooled data of the Hb drop from five studies showed that no statistical differences were observed in terms of favoring oral administration compared with the intravenous administration group (SMD = − 0.03,95%CI = − 0.18–0.12, *P* = 0.67) (Fig. 2).

#### Pooled analysis of total Hb loss between oral administration and intravenous administration

**Fig. 3:**
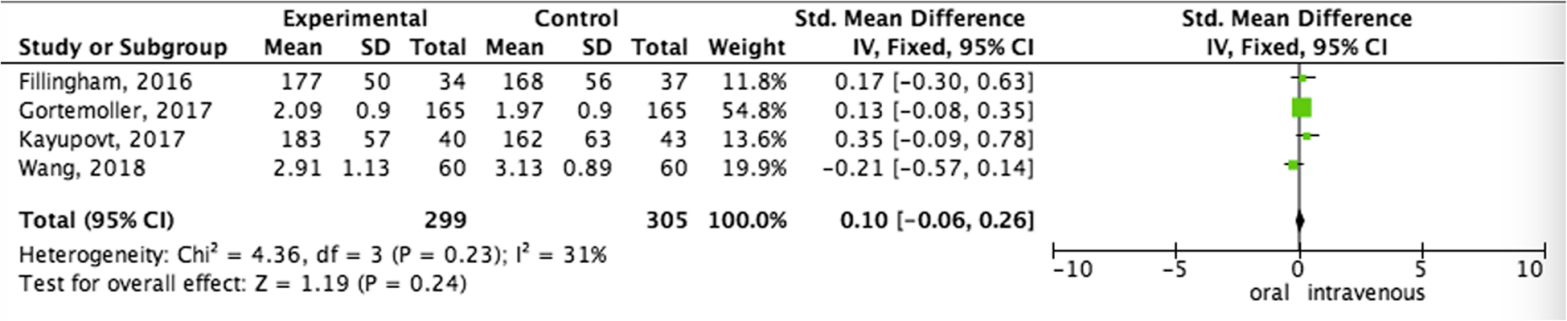
Pooled analysis of total Hb loss between oral administration and intravenous administration

In terms of total Hb loss, no significant differences compared oral administration and intravenous administration was observed (OR = 0.73; 95% CI = 0.51–1.05; *p* = 0.09) (Fig. 3).

#### Pooled analysis of total blood loss between oral administration and intravenous administration

**Fig. 4:**
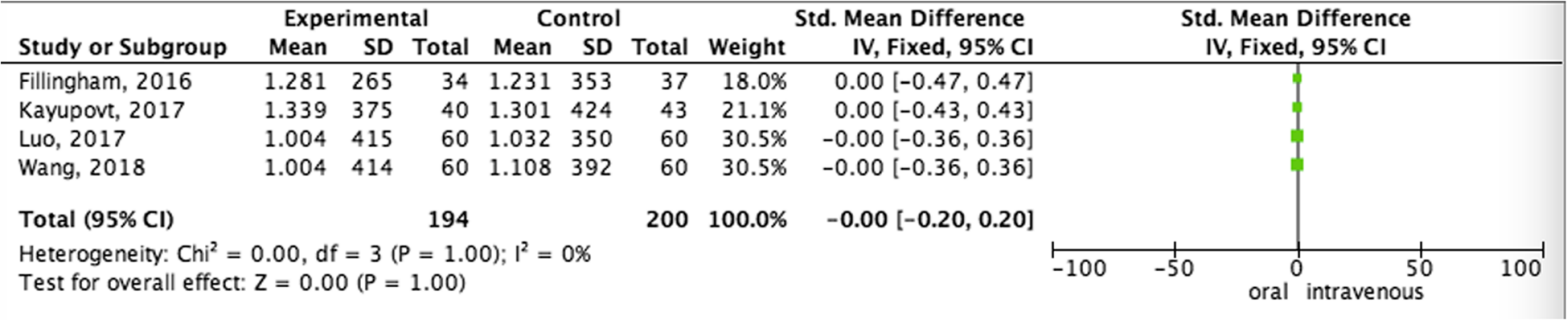
Pooled analysis of total blood loss between oral administration and intravenous administration

Fixed-effect model was used to pool the total blood loss data, since the heterogeneity across the four studies was insignificant. The pooled data showed that oral administration did not increase total blood loss (SMD = − 0.00,95%CI = − 0.20–0.20, *P* = 1.00) than intravenous administration therapy (Fig. 4).

#### Pooled analysis of transfusion rate between oral administration and intravenous administration

**Fig. 5:**
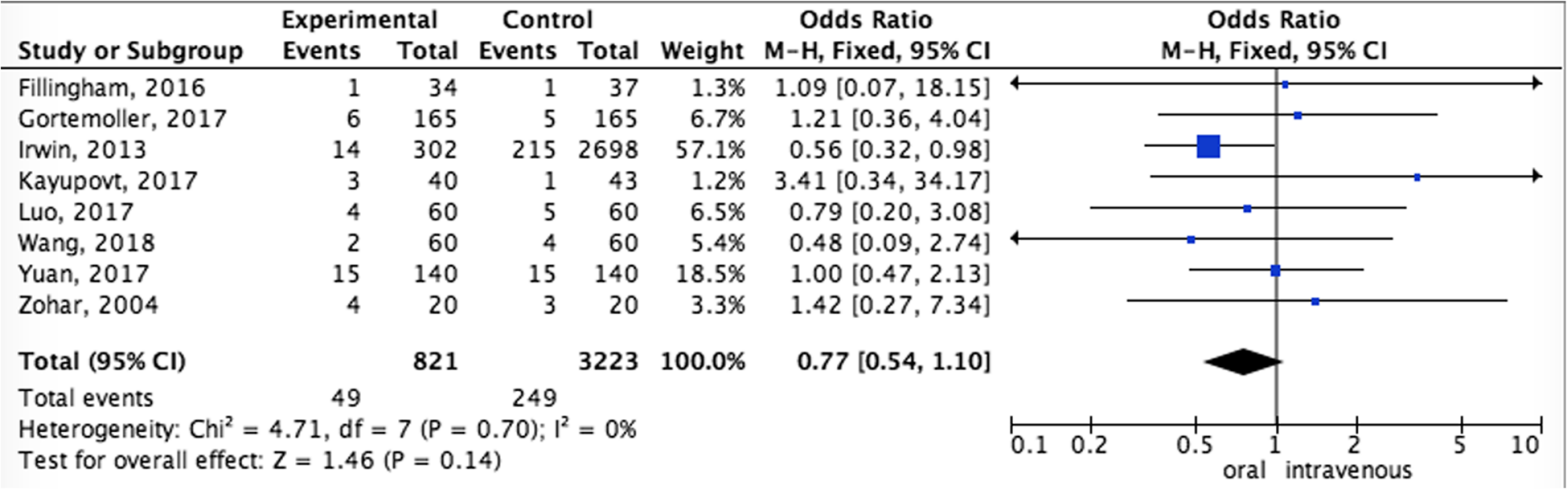
Pooled analysis of transfusion rate between oral administration and intravenous administration

The pooling transfusion rate data did not achieve advantage in the oral administration (OR = 0.77,95%CI = 0.54–1.10, *P* = 0.14). In other words, oral administration compared to intravenous administration did not increase the rate of transfusion (Fig. 5).

#### Pooled analysis of DVT rate between oral administration and intravenous administration

**Fig. 6:**
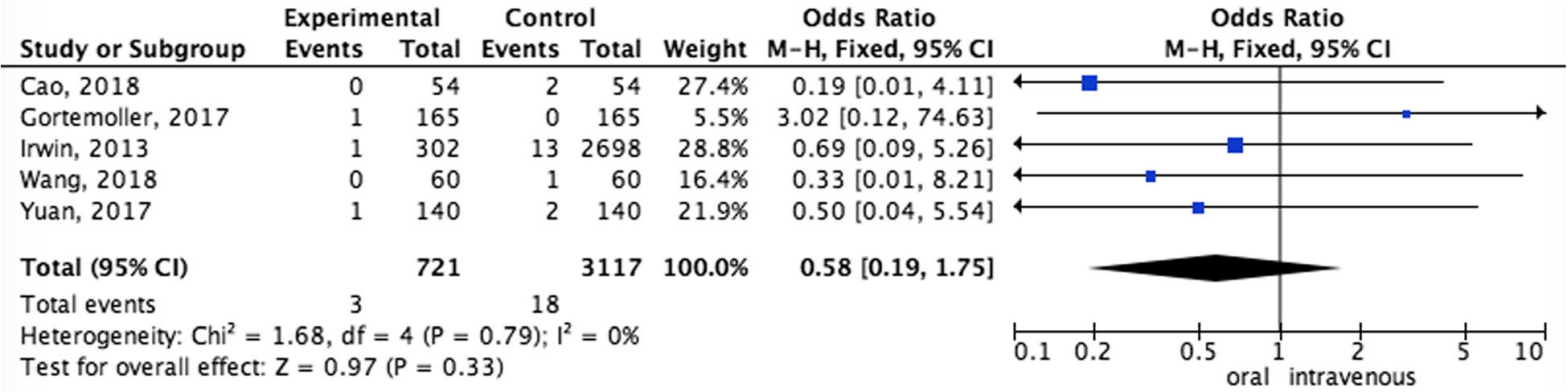
Pooled analysis of DVT rate between oral administration and intravenous administration

The pooled data showed that oral administration did not increase the rate of DVT (OR = 0.58,95%CI = 0.19–1.75, *P* = 0.33) than intravenous administration (Fig. 6).

#### Pooled analysis of length of hospital stay between oral administration and intravenous administration

**Fig. 7:**
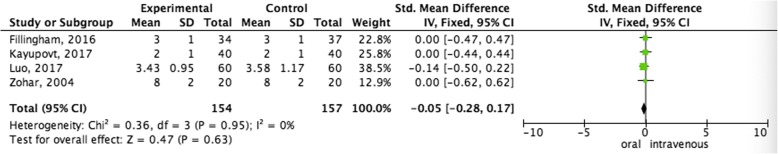
Pooled analysis of length of hospital stay between oral administration and intravenous administration

The pooling analysis revealed that there was no difference in length of hospital stay between oral administration and intravenous administration (SMD = − 0.05,95%CI = − 0.28–0.17, *P* = 0.63) (Fig. 7).

## Discussion

Tranexamic acid has become a widely used to decrease blood loss and transfusion rates following THA and TKA, mainly administrated in the IV in previous studies [8, 24–26]. Compared with intravenous TXA, oral TXA is convenient to administer, reaches cost-benefit advantage, and increases the safety [11, 19]. However, limited research has been done on the use of oral TXA in total joint arthroplasty, and the optimal regimen for TXA has remained uncertain, which have been extensively explored in recent RCTs [27–29]. Our meta-analysis aims to assess the effects of intravenous or topical routes of administration.

The results of this study suggested that no significant differences were found in blood-sparing management between oral and IV TXA. This finding is comparable to the findings of a recent meta-analysis that shown that oral TXA provided equivalent blood-sparing efficacy when compared with intravenous routes [23].

The Hb drop accounted for > 60% of total blood loss in total joint arthroplasty [30, 31]. Due to the blood loss may leading to postoperative hypoxemia, the blood transfusion is required. However, blood transfusion is associated with adverse effects and increases the risk of complications resulting from hypervolemia [32–34]. Hence, rational and effective method for minimizing perioperative blood loss is needed. Previous studies have reported that IV-TXA has beneficial effects on blood-sparing, which has been widely used in patients undergoing both TKA and THA. However, IV administration of TXA has increased the risk of anaphylactic reaction [35]. Moreover, specific equipment is required for IV administration of TXA, which prolong the surgery time.

Recently, some RCTs have demonstrated that oral TXA administration was non-inferior to IV administration in the prevention of perioperative blood loss [11, 18, 19]. Kayupov et al. [18] found that no difference in the reduction of Hb was seen between two groups. Coincidentally, both Yuan et al. [19] and Fillingham et al. [11] have reported the same result. Moreover, these authors further reported that oral TXA meet similar with the IV TXA in terms of the total Hb loss following TKA and THA. Our meta-analysis was consistent with these results.

In terms of the transfusion rate, a RCT carried out by Kayupov et al. [18] found no significant difference between the two routes of administration following THA. Yuan et al. [19] compared the transfusion rate between oral administration and IV administration in patients who received TKA, and this difference was not statistically significantly different. This result was consistent with our findings.

DVT is a common postoperative complication, which may promote the risk of death in TKA and THA [36]. TXA has been reported to be associated with a potential higher risk of thrombotic complication, which might be due to the tendency of DVT, and plays as anti-fibrinolytic agent to promote the risk of clotting. The intravenous administration might be more likely to cause thrombus due to the higher TXA level of blood concentration. Our study found that the two routes of administration of TXA were similar in terms of the risk of DVT.

Admittedly, there were a few limitations in the current study that should not be ignored. First, the main strength of this article is the use of a well-maintained and updated database. Nevertheless, due to all included studies’ retrospective nature, bias still exist, and this may impact the comparison of clinical outcomes. Second, several potential variations, such as hemodilution, dosing strategies and timing of oral TXA, may affect estimated blood loss [20]. However, these variations provided insufficient data. Thus, there was no strong statistical evidence to analyze. Thirdly, the cost is another important evidence to help to inform decision-making when choosing the standard treatment option of TXA. The therapeutic attempts are justifiable if the better efficacy as well as cost savings can be achieved.

## Conclusion

Oral TA was non-inferior to IV TA for blood-sparing efficacy without increasing the risk of thromboembolic diseases in THA and TKA. The efficiency, safety, and cost are considered to be crucial parameters during the decision-making of TXA routes, whether oral TXA can stands as an optimal administration of reducing blood loss following the THA and TKA compared with the IV and other forms still remains a matter of debate in future.
